# Towards inclusive healthcare: evaluating knowledge, confidence and awareness of LGBTQ + health among Internal Medicine Trainees in London

**DOI:** 10.1186/s12909-024-05827-y

**Published:** 2024-08-07

**Authors:** Andrew Crowe, Patrick Hogan, Christoper Morrison, Catherine Meads, Daniel Bailey

**Affiliations:** 1 Faculty of Health, Medicine and Social Care, Anglia Ruskin University, Cambridge, UK; 2 St Pancras Hospital, Central and North West London NHS Foundation Trust, London, UK; 3 Public Health Directorate, NHS Grampian, Aberdeen, UK; 4 Kings College Hospital NHS Foundation Trust, London, UK

**Keywords:** LGBT, Medical education, Postgraduate, Sexual orientation, Gender identity, Internal medicine, Training

## Abstract

**Background:**

Patients from the lesbian, gay, bisexual, transgender, queer plus (LGBTQ +) community face various health inequalities and report poor healthcare experiences. Little is known about how knowledgeable and confident UK doctors are around LGBTQ + health, and previous research demonstrates that UK medical schools rarely deliver teaching in this area. This research evaluated the level of knowledge, awareness and confidence of LGBTQ + health among Internal Medical Trainees (IMTs) in London.

**Methods:**

London IMTs were invited to complete an online questionnaire evaluating knowledge, awareness and confidence in LGBTQ + health. Stratified analysis of results by demographics was performed.

**Results:**

Three hundred and fifteen surveys were analysed from 796 eligible trainees (40%). Confidence in caring for LGBTQ + patients was variable. Confidence in discussing gender identity was lower than for sexual orientation. Knowledge of health issues affecting LGBTQ + patients varied. Most participants had never received training on LGBTQ + health at undergraduate (*n* = 201, 64%) or postgraduate level (*n* = 252, 80%), but the majority of participants felt that training would be useful (*n* = 233, 74%). Stratified analysis revealed that IMTs who received previous LGBTQ + teaching at undergraduate or postgraduate level were considerably more confident discussing sexual orientation with patients, compared to those who received no previous teaching.

**Conclusions:**

There is a clear need for education on LGBTQ + health, given the varied levels of knowledge and confidence identified. A significant majority of IMTs in London have never received teaching on LGBTQ + health, although there exists a strong desire for this. LGBTQ + health topics should be integrated into undergraduate and postgraduate training and examinations for IMTs. This would support IMTs in delivering high quality and inclusive care for all patients, particularly those of sexual orientation and gender identity minorities. There are relatively few published studies exploring competency in LGBTQ + health among doctors, and this is the first among UK Internal Medicine Trainees.

## Background

In recent times, the spotlight on healthcare disparities faced by marginalised communities has grown stronger, and the voices of these communities have grown louder [1]. LGBTQ + communities are one such marginalised group, composed of people who are Lesbian, Gay, Bisexual, Transgender, or Queer. The “plus” denotes people who are part of the community, but for whom LGBTQ + neither accurately captures, nor reflects their identity. They frequently report negative encounters in the healthcare setting and experience unique health inequalities in areas such as physical health, sexual health, and mental health [2].

Cancer burden is greater in the LGBTQ + communities, with higher rates of anal cancer among men who have sex with men [3] and higher rates of cervical intraepithelial neoplasia among women who have sex exclusively with women [4]. In addition, lesbian and bisexual women in the UK have higher rates of asthma and obesity compared to heterosexual women [5, 6]. Transgender individuals are significantly more likely to be living with chronic medical and psychiatric conditions (including dementia) and have suicide rates at least 5 times higher than their cisgender peers [7, 8]. LGBTQ + patients of nearly all age groups are more likely to avoid seeing their GP, contributing to late diagnosis and poor outcomes [9].

One potential factor contributing to these health inequalities is the ‘Minority stress theory’, which suggests that LGBTQ + people experience chronic stress from both “distal” sources (e.g. discrimination, victimisation, bullying, stigmatisation, violence, and social injustice), and “proximal” sources (e.g. internalised homophobia and perceived prejudice) [10]. This chronic stress response may lead to increased risk of various physical health conditions, mental health conditions including suicidality, and increases the likelihood of engaging in high-risk and harmful behaviours [11, 12]. Among transgender people, negative physical health outcomes were actually more common in those with past experiences of significant harassment or violence, compared to those without [13]. Through education, clinicians can become aware of the Minority stress theory, and actions that can potentially contribute to this either overtly (e.g. through expression of prejudicial opinions), or inadvertently (e.g. by using heteronormative and cisnormative language, by providing services that do not appear inclusive).

Another factor potentially contributing to health inequalities is engagement with healthcare. LGBTQ + people may have concerns about disclosing sexual orientation/gender identity to healthcare providers, based on previous experience of discrimination, the perception or fear of it, and concerns that services will neither understand, nor support their needs [14]. As a result, they may not engage with screening programmes or seek help for concerning symptoms, leading to missed opportunities for cancer detection, or primary/secondary prevention of disease [2]. For example, women who have sex exclusively with women are less likely to attend for cervical cancer screening [4], and as previously stated, they have higher rates of cervical intraepithelial neoplasia (a precursor to cervical cancer). Transgender people report higher rates of negative experiences in healthcare, and are more likely to avoid seeking care than their cisgender counterparts [15]. Studies demonstrate they have increased rates of chronic medical conditions, and poor mental health, particularly suicidal ideation [7, 8].

LGBTQ + communities have contrasting experiences of health and healthcare compared to the general population. Current and future clinicians should be cognisant of these differences and their role in addressing them.

The area of LGBTQ + health remains understudied and under-researched; it is not widely covered in curricula of UK medical schools. For many medical schools, there is little or no exposure to LGBTQ + teaching during the undergraduate programmes [16, 17]. Medical students feel unprepared for encounters with LGBTQ + patients, which could translate into poor quality of care [18, 19]. Inclusion and cultural competence are increasingly recognised to be important in healthcare, and this knowledge gap may contribute to suboptimal care, and worsen health disparities experienced by LGBTQ + individuals. With increasing numbers of people identifying as LGBTQ + , doctors must be competent to provide care to patients from these communities [20].

There is a dearth of literature describing LGBTQ + health in medical education and little is known about the knowledge and confidence of UK clinicians around these issues. The vast majority of published literature in this area focuses on the undergraduate setting and explores how confident and knowledgeable medical students are, or evaluates the amount of LGBTQ + teaching in undergraduate curricula [16, 17]. In relation to medical graduates (i.e. qualified doctors), there are very few published studies and only one other in the British setting which focuses solely on Oncologists [21], making this the first study of its kind among IMTs in the United Kingdom.

The core aim of this study was to evaluate the levels of knowledge, confidence, and awareness that Internal Medicine Trainees (IMTs) in London have around the health needs of patients from the LGBTQ + community. Our objectives included: assessing how confident IMTs feel when caring for patients from this community, examining how knowledgeable IMTs are in LGBTQ + health, determining how much prior teaching IMTs have received on LGBTQ + health and how useful they feel specialist teaching would be, and investigating the demographics of participants in a stratified analysis.

In this article, we use the terms MSM (men who have sex with men) and WSW (women who have sex with women). These terms describe sexual activities without assuming identities like gay, lesbian, or bisexual, recognising that not everyone who engages in same-sex activities identifies with these labels.

Through this research, we identify areas for improvement, and consequently, provide the evidence needed to design targeted interventions and implement curricular changes that could equip future doctors with the skills to confidently care for this marginalised and vulnerable population group.

## Methods

### Study design

We designed and conducted an observational cross-sectional study with mixed quantitative/qualitative methods. Our core research question was: What is the level of awareness, confidence and knowledge in LGBTQ + health among IMTs in London?

We included all 796 IMTs (years 1–3) currently training in a London Deanery. IMTs are qualified doctors who have completed Foundation Training and have chosen to train in Internal Medicine (they are at least 2 years after graduation). After completion of Internal Medicine training, the majority will enter specialist medical training (in Cardiology, Gastroenterology, Neurology, etc.).

We identified IMTs for inclusion as they form a large and accessible cohort of doctors, thus providing a suitable sample size. In addition, they interact with patients on a daily basis and are likely to encounter members of LGBTQ + community in a professional context. We focused on London as it has the largest proportion of LGBTQ + residents in the United Kingdom [22].

### The survey

The online questionnaire was designed using Jisc software, a program for designing and distributing online surveys. The surveys were emailed to participants four times over a 2-month period via the London School of Medicine. These questionnaires were self-administered by participants, and participation was voluntary. Consent was compulsory in order to complete the questionnaire and participants were asked to read the Participation Information Leaflet and tick the consent box if in agreement. The participants were not asked for personally identifiable information such as name, date of birth or address, but were asked to provide some demographic details. There was a "prefer not to say" option for each demographic question.

There were 33 questions, in 5 sections. The majority were closed questions with true/false or yes/no answers. Other question formats included multiple choice questions, Likert scale questions and free text boxes for comments or feedback.

The first section assessed demographics, the second section explored levels of awareness and confidence in caring for LGBTQ + patients, the third section assessed prior teaching on LGBTQ + health received by participants, the fourth section examined knowledge of LGBTQ + health and the fifth section asked for comments and feedback. The correct answers to each question in the knowledge section, along with an explanation and reference to the literature, were provided upon completion of the survey to promote learning for all participants.

These questions were designed to focus on scenarios encountered by IMTs, thus making the survey directly relevant to their practice. A pilot questionnaire was completed by a small group of IMTs, and questions were refined based on their feedback. We concentrated on general internal medicine, an area often neglected in LGBTQ + health research, rather than other areas such as sexual health. We designed 3 separate question stems to individually test knowledge on gay male health, gay female health, and trans health. Due to limited survey space, we were unable to include as many identities as we wished (bisexual, non binary etc.).

### Data analysis

Every survey answered was used in data analysis, which was done with SPSS software and descriptive analysis of the data. Data was presented in graphs and charts made using Microsoft Excel. In certain demographic questions and other parts of the results where fewer than five respondents answered, the results are reported in text and tables as < 5 in order to promote confidentiality and reduce risk of participant identification.

Ethical Approval was granted by the School Research Ethics Panel (SREP) of the Health, Education, Medicine and Social Care (HEMS) faculty of Anglia Ruskin University.

## Results

There were 315 responses (40% of the total eligible population). All surveys were fully completed. Most respondents were aged 26-30yrs. (*n* = 198, 62.9%), and slightly more participants were female, with 160 female participants (50.8%), 140 male participants (44.4%), and the rest indicating 'prefer not to say' (*n* = 15, 4.8%). 23.1% of participants identified as LGBTQ + , with 6.7% ticking "prefer not to say" for sexual orientation, and 5.7% for gender identity. For demographics—See Table 1.

**Table 1:** Participant demographics

Characteristic	Categories	Number (Percentage)
Age:	26–30	198 (62.9%)
	31–35	96 (30.5%)
	36–40	10 (3.2%)
	41 +	< 5 respondents
	Prefer not to say	< 5 respondents
Gender:	Male	140 (44.4%)
	Female	160 (50.8%)
	Prefer not to say	15 (4.8%)
Gender identity:	Cisgender	291 (92.4%)
	Transgender	< 5 respondents
	Non-binary	5 (1.6%)
	Other	< 5 respondents
	Prefer not to say	18 (5.7%)
Sexual orientation:	Straight	225 (71.4%)
	Gay	38 (12.1%)
	Bisexual	26 (6.7%)
	Other	5 (1.6%)
	Prefer not to say	21 (6.7%)
Place of training:	North London	171 (54.3%)
	South London	132 (41.9%)
	Prefer not to say	12 (3.8%)
Religion:	Christian	58 (18.4%)
	Islam	19 (6%)
	Hinduism	12 (3.8%)
	Sikhism	5 (1.6%)
	Judaism	5 (1.6%)
	Buddhism	< 5 respondents
	Atheist	117 (37.1%)
	Agnostic	46 (14.6%)
	Prefer not to say	44 (14%)
	Other	5 (1.6%)
Level of religiousness:	Strongly religious	10 (3.2%)
	Somewhat religious	57 (18.1%)
	Not religious	106 (33.7%)
	Atheist/Agnostic	113 (35.9%)
	Prefer not to say	29 (9.2%)
Attended medical school in:	UK	272 (86.3%)
	Europe (excluding UK)	27 (8.6%)
	North America	1 (0.3%)
	South America	1 (0.3%)
	Asia	10 (3.2%)
	Africa	2 (0.6%)
	Oceania	0 (0%)
	Other	2 (0.6%)

### Confidence/awareness

When asked about confidence in discussing issues of sexual orientation and gender identity with patients (See Table 2), responses varied, but confidence levels around gender identity were lower than sexual orientation. Just over half of participants (54.3%) felt confident asking a patient about sexual orientation, while 27.6% did not feel confident, and 18.1% felt somewhat confident. Regarding gender identity, 45.1% of participants felt confident asking patients about gender identity, 33.3% did not feel confident, and 21.6% felt somewhat confident. Less than half (46.0%) felt confident using terms related to gender identity (pronouns, transgender, non-binary etc.), while 30.8% did not feel confident, and 23.2% felt somewhat confident. When asked if participants had ever treated patients who identified as LGBTQ + , 289 respondents (91.7%) replied Yes, 12 participants (3.8%) replied No, and 14 (4.4%) were not sure.

**Table 2:** Confidence and awareness

Question	Categories	N (%)
Do you feel confident asking a patient about their sexual orientation if you thought it was relevant?	**Yes**	**171 (54.3%)**
	**No**	**87 (27.6%)**
	**Sometimes**	**57 (18.1%)**
Do you feel confident asking a patient about their gender identity if you thought it was relevant?	**Yes**	**142 (45.1%)**
	**No**	**105 (33.3%)**
	**Sometimes**	**57 (18.1%)**
Do you feel confident using terms related to gender identity? (pronouns, transgender, non binary etc.)	**Yes**	**145 (46%)**
	**No**	**97 (30.8%)**
	**Sometimes**	**73 (23.2%)**
To your knowledge, have you ever treated patients who identify as LGBTQ + ?	**Yes**	**289 (91.7%)**
	**No**	**12 (3.8%)**
	**Unsure**	**14 (4.4%)**
Do you feel that knowing whether a patient identifies as LGBTQ + is important when providing medical care?	**Yes**	**131 (41.6%)**
	**No**	**28 (8.9%)**
	**Sometimes**	**156 (49.5%)**

### Training

Most participants reported having no prior exposure to training on LGBTQ + health, (See Table 3), a slightly greater proportion of participants received LGBTQ + training during their undergraduate training than during postgraduate training (36.1% during undergraduate vs 20.0% during postgraduate). A large proportion felt that LGBTQ + teaching was useful: 233 participants (73.9%) felt it was "very useful", 79 participants (25.1%) felt it was "somewhat useful", and 3 participants (0.9%) felt it was "not useful". Participants were keen for teaching on various areas of LGBTQ + health but particularly on the topics of general medicine in LGBTQ + patients (85.4%) and transgender healthcare (66.7%).

**Table 3:** Teaching on LGBTQ + health

Question	Categories	N (%)
Did you receive any formal LGBTQ + health teaching during your medical undergraduate degree program? (university)	**None**	**201 (63.8%)**
	**Few hours**	**46 (14.6%)**
	**1 h**	**36 (11.4%)**
	**Few days**	**18 (5.7%)**
	**1 day**	**14 (4.4%)**
Have you received any formal LGBTQ + health teaching since you graduated from medical school?	**None**	**252 (80%)**
	**Few hours**	**27 (8.6%)**
	**1 h**	**30 (9.5%)**
	**Few days**	**4 (1.3%)**
	**1 day**	**2 (0.6%)**
Do you believe that teaching on LGBTQ + health is useful for IMTs?	**Very useful**	**233 (74%)**
	**Somewhat useful**	**79 (25.1%)**
	**Not useful**	**3 (1%)**
What area of LGBTQ + health would you most like to receive teaching on? Tick all that apply	**General medicine for LGBTQ + patients**	**269 (85.4%)**
	**Sexual health in LGBTQ + patients**	**192 (61%)**
	**Transgender healthcare**	**210 (66.7%)**
	**Mental health in LGBTQ + patients**	**141 (44.8%)**
	**Health in older LGBTQ + patients**	**177 (56.2%)**
	**Cancer care for LGBTQ + patients**	**157 (49.8%)**
	**Other**	**2 (0.6%)**

### Knowledge

**Fig. 1:**
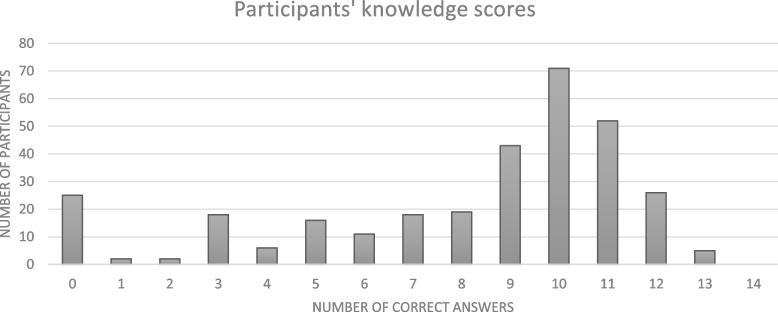
Distribution of knowledge scores

Distribution of knowledge scores was varied (See Table 4 and Fig. 1). Below are some pertinent results from the knowledge section:

- When asked about rates of asthma and average BMI in lesbian women, most answers were incorrect (90.8% incorrect and 72.1% incorrect respectively)
- 64.8% of respondents correctly identified that lesbian women in the UK do not have higher rates of cardiovascular disease compared to the general populations, and 60.6% correctly recognised that nulliparity is a risk factor for breast cancer in lesbian women (as for all nulliparous women)
- 72.1% correctly identified that men who have sex with men (MSM) are more likely to develop anal cancer than heterosexual men. However, over one third (34.0%) incorrectly believed they are more likely to develop colon cancer, compared to heterosexual men.
- 65.0% of respondents correctly answered than older gay men are twice as likely to be living alone compared to older heterosexual men.
- 67.9% correctly answered that older LGBTQ + individuals are less likely to attend their GP than non-LGBTQ + individuals.
- A minority of respondents (41.0%) correctly answered that rates of Subjective Cognitive Decline (SCD) are higher among LGBTQ + individuals.

**Table 4:** Knowledge

Correct answers (bold text in left column)	Incorrect answers (light text in right column)
**Scenario 1** A 69 year old woman who identifies as lesbian is admitted medically for investigation of shortness of breath and chest tightness. She has no past medical history. She smokes 5 cigarettes per day but wants to stop. She lives alone and has no children. She reports she has not seen her GP in over 20 years. On auscultation of her chest, there is a diffuse faint wheeze. When examining her, she mentions that she noticed a lump in her left breast last month	
Q10. Lesbian women in the UK have higher rates of asthma, compared to population average	
** True 29 (9.2%)**	False 286 (90.8%)
Q11. Lesbian women in the UK have higher rates of cardiovascular disease, compared to the general population	
**False 204 (64.8%)**	True 111 (35.2%)
Q12. Lesbian and bisexual women in UK have a higher average BMI than heterosexual women	
**True 88 (27.9%)**	False 227 (72.1%)
Q13. Nulliparity is a risk factor for breast cancer in women who identify as lesbian	
**True 191 (60.6%)**	False 124 (39.4%)
Q14. Older LGBTQ patients are less likely to attend their GP than older non-LGBTQ patients?	
**True 214 (67.9%)**	False 101 (32.1%)
**Scenario 2** A 76 year old man who identifies as gay is admitted by the medical team with confusion, and weight loss. He lives alone and has no carers. He reports his memory has been worsening for the "last while". He often loses things at home and sometimes forgets where he is. He was unable to give a next of kin, saying he is estranged from his family and he has no close friends as many died of AIDS. He mentions to you that he has noticed some bleeding from his "back passage" for the last number of months	
Q15. Rates of Subjective Cognitive Decline (SCD) are higher in LGBTQ + patients compared to the general population	
**True 129 (41.0%)**	False 186 (59.0%)
Q16. Rates of dementia are higher in LGBTQ + patients, compared to the general population	
**False 194 (61.6%)**	True 121 (38.4%)
Q17. Older gay men are twice as likely to be living alone than older heterosexual men	
**True 207 (65.7%)**	False 108 (34.3%)
Q18. Men who have sex with men (MSM) are more likely to develop anal cancer than heterosexual men	
**True 227 (72.1%)**	False (27.9%)
Q19 Men who have sex with men are more likely to develop colon cancer than heterosexual men	
**False 208 (66.0%)**	True 107 (34.0%)
**Scenario 3** Caleb, a 37 year old transgender man, presents to Urgent Care with abdominal and pelvic pain which started around 2 days ago. He reports he began hormonal therapy with Testosterone injections 9 months ago and says he became amenorrhoeic soon after. He has not has surgical therapy. He also reports headaches and tiredness for the last number of weeks. Blood work reveals the following: Hb 178, Hematocrit 0.61, WCC 11.2, platelets 332, CRP 61, creatinine 52, EGFR > 90	
Q20. A prostate examination should be considered to assess for acute prostatitis	
**False 210 (66.7%)**	True 105 (33.3%)
Q21. Checking Urine B-HCG level should be considered to assess for pregnancy or ectopic pregnancy	
**True 220 (69.8%)**	False 95 (30.2%)
Q22. He may be at increased risk of ischemic stroke and myocardial infarction	
**True 225 (71.4%)**	False 90 (28.6%)
Q23. The testosterone therapy should be discontinued immediately	
**False 211 (67.0%)**	True 104 (33.0%)
**Mean of correct answers**	Mean of incorrect answers
**182, 57.9%**	133, 42.0%

### Stratified analysis

Stratified analysis (See Table 5) revealed that the participants who received previous LGBTQ + teaching at undergraduate or postgraduate level were considerably more confident discussing sexual orientation with patients, compared to those who received no previous teaching (statistically significant) These participants were also more confident in discussing gender identity with patients – this was statistically significant for participants who received teaching at undergraduate level, but not for those who received teaching at postgraduate level. Males felt slightly more confident discussing sexual orientation and gender identity with patients compared to females (not statistically significant). IMTs with prior teaching were more likely to feel that knowing a patient’s sexual orientation or gender identity is important when caring for them, compared to those who with no prior training (statistically significant).

**Table 5:** Stratified analysis

		No under-graduate training	Some under-graduate training	No post-graduate training	Some post-graduate training	Self-identify as straight	Self-identify as Gay or Bisexual or other	Prefer not to say	Self-identify as cisgender	Self-identify as Trans-gender or Nonbinary or other	Prefer not to say	Male	Female	Prefer not to say
Do you feel confident asking about Sexual orientation?	YES	95 (47.2%)*	76 (66.6%)*	129 (51.6%)*	42 (66.7%)*	111 (49.3%)*	49 (71%)*	11 (52.4%)*	161 (55.3%)	3 (50.0%)	7 (38.9%)	85 (60.7%)	80 (50.0%)	**6 (40.0%)**
	NO	71 (35.3%)*	16 (14.0%)*	77 (30.7%)*	10 (15.9%)*	68 (30.2%)*	10 (14.5%)*	9 (42.9%)*	77 (26.5%)	1 (16.7%)	9 (50.0%)	30 (21.4%)	51 (31.9%)	**6 (40.0%)**
	Sometimes	35 (17.4%)*	22 (19.2%)*	46 (18.3%)*	11 (17.5%)*	46 (20.4%)*	10 (14.5%)*	1 (4.8%)*	53 (18.2%)	2 (33.3%)	1 (11.1%)	25 (17.9%)	29 (18.1%)	**3 (20.0%)**
Do you feel confident asking about Gender identity?	YES	80 (39.8%)*	62 (54.4%)*	110 (43.7%)	32 (50.8%)	89 (39.6%)*	43 (62.3%)*	10 (47.6%)*	132 (45.4%)	3 (50.0%)	7 (38.9%)	67 (47.9%)	68 (42.5%)	**7 (46.7%)**
	NO	83 (41.3%)*	22 (19.3%)*	88 (34.9%)	17 (27.0%)	81 (36.0%)*	15 (21.7%)*	9 (42.9%)*	94 (32.3%)	2 (33.3%)	9 (50.0%)	44 (31.4%)	54 (33.8%)	**7 (46.7%)**
	Sometimes	38 (18.9%)*	30 (26.3%)*	54 (21.4%)	14 (22.2%)	55 (24.4%)*	11 (15.9%)*	2 (9.5%)*	65 (22.3%)	1 (16.7%)	2 (11.1%)	29 (20.7%)	38 (23.8%)	**1 (6.7%)**
Is it important to know if a patient is LGBTQ + when caring for them?	YES	87 (43.3%)*	44 (38.6%)*	102 (40.5%)*	29 (46.0%)*	83 (36.9%)	37 (53.6%)	11 (52.4%)	120 (41.2%)	4 (66.7%)	7 (38.9%)	56 (40.0%)	67 (41.9%)	**8 (53.5%)**
	NO	26 (12.9%)*	2 (1.8%)*	27 (10.7%)*	1 (1.6%)*	22 (9.8%)	3 (4.3%)	3 (14.3%)	25 (8.6%)	0 (0.0%)	3 (16.7%)	14 (10.0%)	12 (7.5%)	**2 (13.3%)**
	**Sometimes**	88 (43.8%)*	68 (59.6%)*	123 (48.8%)*	33 (52.4%)*	**120 (53.3%)**	**29 (42.0%)**	**7 (33.3%)**	**146 (50.2%)**	**8 (44.4%)**	**8 (44.4%)**	**70 (50.0%)**	**81 (50.6%)**	**5 (33.3%)**

Stratified analysis was done using SPSS and crosstabulations with Pearson Chi-square testing

The statistically significant values (*p* < 0.05) are in bold and followed by an asterisk (*)

‘Some undergraduate training’ denotes: 1 h, few hours, 1 day or few days

‘Some postgraduate training’ denotes: 1 h, few hours, 1 day or few days

### Feedback

Participants were invited to give feedback in two free text boxes (See Table 6). The first box asked if LGBTQ + teaching was worthwhile and how should it be done. The second box asked for any further comments or feedback. There were 113 responses in total. Some commonly occurring themes were;

**Table 6:** Feedback and Final Comments (a selection of 113 responses in total)

We would love to hear your thoughts on LGBTQ + health and teaching—Is it worthwhile?
Do you have any final comments or feedback?
We grouped the feedback into four themes:
1) Desire for teaching, particularly on trans healthcare and general internal medical issues in LGBTQ + patients
• I have looked after patients who identify as LGBTQ + and have felt ill-equipped to manage this well. Since these experiences I have tried to look up the correct terminology and language to use but it is still not an area of confidence for me, and I do not know much about the impact of this on general medicine for this patient cohort. Any teaching would be gratefully received
• It is a large and poorly understood minority, prone to significant stereotypes that are unhelpful and discriminatory. My only LGBTQ + "teaching" in my degree was essentially being encouraged to assume HIV/HIV-related disease as a diagnosis any question where it mentions the patient as being homosexual/MSM in the stem. As a gay man myself I found this pretty insulting
• A session would be good. Maybe even include LGBTQ + scenarios in paces. I found paces exam far too gender stereotyped and traditional scenarios
• I think teaching in this is essential!! Medical training is very discriminatory, and all we really get taught in medical school is that gay men in exam questions always have HIV
• We need teaching on this. I think that teaching on LGBT Health is still mostly about HIV and STIs in MSM. I think in particular it would be useful to have teaching on trans healthcare (including the process of transitioning in the UK, hormone treatments and their potential complications). Information about chem sex would also be useful e.g. common drugs and spotting and managing overdose, and no one seems to understand PrEP so that would be useful too
• I think LGBTQ + health and well-being are very neglected parts of the undergraduate and postgraduate curriculum. I myself would love to be a part of improving this and bringing about change but simply don’t know where to start. I think there should be more awareness of LGBTQ + concepts definitions and appreciation of recognising the LGBTQ + patient in order to better provide more personalised care
• I would like clarity on terms. eg I try to refer to a transgender woman as female, with it clearly documented in notes that the patient is biologically male, as this has implications for them receiving the best care. Understand this could be upsetting for them—how can I approach this in a way that satisfies my medical obligations and doesn't damage patient-doctor relationship
• It is worthwhile and should be incorporated as part of the curriculum and not an option for those who are interested. There should be a much higher baseline knowledge of how to best treat LGBTQ patients for IMTs and the profession as a whole. Medicine is not immune from homophobic attitudes or transphobic attitudes and normalising teaching about LGBT peoples helps tackle this
• This is certainly a group that is missing from current medical education and I think certain risk factors are not at the forefront of our brain when history taking. Informal group discussion/tutorials would be useful to discuss how to ask certain questions. Case studies such as those above are great
• Case studies really interesting in this quiz and flagged lots of general medical areas I would not have considered
2) Authenticity of teaching,
• Teaching would be very worthwhile. Ideally teaching should be delivered in-person by people of the LGBTQ + community so that they are not misrepresented and we can hear patient’s perspectives
• It’s difficult. I’d like older generations of doctors to have an appreciation of LGBTQ + issues and for us to learn from positive experiences but I don’t see this happening. Honestly personal experiences from an LGBTQ + patient would probably be the most impactful type of teaching
• I have had previous teaching which involved members of the LGBTQ + community sharing their healthcare experiences and what should have been done differently—I would like more of this
3) Negative experiences while working
• Have seen some really transphobic and homophobic stuff working in the NHS and so we definitely need more education and open dialogues about LGBTQ health. Ideally it should be integrated into all our teaching, as should women's health e.g. in the regional IMT teaching sessions speakers could be asked to give slide per topic on how this disease affects everyone who is not a 70 kg cis white man
• Cultural background has a strong influence on knowledge and attitudes to LGBTQ + individuals and I have witnessed higher rates of transphobia from colleagues from other cultures which directly affected patient care and outcomes
4) Other:
• As with all specialist teaching in IMT, there is definitely a benefit to IMTs understanding more about LGBTQ + health, however this needs to be carefully balanced against other learning needs and I would like to see stats on patients coming to harm because of doctors' lack of knowledge about LGBTQ + conditions versus lack of knowledge about other clinical conditions before new teaching is implemented. In short, I think teaching needs to be governed by clinical need
• Should not be the priority, but I believe that at least a couple of sessions per year should be attended

1. Desire for teaching, particularly on trans healthcare and general internal medical issues in LGBTQ + patients:
  “I have looked after patients who identify as LGBTQ+ and have felt ill-equipped to manage this well. Since these experiences I have tried to look up the correct terminology and language to use but it is still not an area of confidence for me, and I do not know much about the impact of this on general medicine for this patient cohort. Any teaching would be gratefully received”
2. Authenticity of teaching,
  “Ideally teaching should be delivered in-person by people of the LGBTQ+ community so that they are not misrepresented and we can hear patient’s perspectives”
3. Negative experiences while working.
  “Have seen some really transphobic and homophobic stuff working in the NHS and so we definitely need more education and open dialogues about LGBTQ health”

## Discussion

### Summary of findings

Overall, this study reveals that knowledge levels around LGBTQ + health among IMTs in London are varied. They are moderately confident discussing sexual orientation with patients, but less confident discussing gender identity and its related terminology (transgender, non-binary, pronouns etc.). Most participants have never received any formal teaching on LGBTQ + health, which is consistent with the literature showing these topics are rarely covered at undergraduate or postgraduate level [16, 17]. However, it is encouraging to see there is a strong demand for this, particularly teaching on general medicine for LGBTQ + patients and transgender healthcare.

Our results compare similarly to findings from two American studies [23, 24]. In both studies, IMTs felt that LGBTQ + health was important, but they reported minimal prior teaching in this area and assessment of their knowledge revealed numerous deficits. Confidence levels were varied but increased after teaching.

A significant proportion of the surveyed IMTs felt under-confident discussing sexual orientation and gender identity with patients. Of note, participants were less confident discussing gender identity (and related terms such as transgender, non-binary and pronouns) than sexual orientation. One third of participants were not confident asking patients about gender identity. Stratified analysis revealed that participants who had received previous formal LGBTQ + training (at undergraduate or postgraduate level) reported higher levels of confidence in these areas compared to those who never received teaching, demonstrating the benefits of teaching, and reinforcing the need for formal education. Of note, participants who received training during university reported feeling more confident than those who did not. Although causation cannot be assumed, these findings suggest the effect of training in improving confidence may last for several years (at least 3 years in the case of this cohort of IMTs).

The proportion of surveyed participants identifying as gay (12.1%), bisexual (8.3%) or other (1.6%) was higher than the proportion in the general population. In the 2021 UK Census [17], 4.3% of London residents identified as lesbian, gay, bisexual, or other. Our figures could be explained by the younger age group of IMT participants (93.4% of participants were in the 26–35 age bracket) who are statistically more likely to identify as LGBTQ + than older age groups [22]. Additionally, these figures could reflect the potential responder bias associated with voluntary participation in surveys – for example, people identifying as gay, or bisexual are more likely to voluntarily take surveys about LGBTQ + issues. Regarding gender identity, just 0.3% of participants identified as transgender, and 1.6% as non-binary, which compares slightly differently to the general population of London residents where 0.78% identify as transgender/gender different from that assigned at birth, and 0.8% identify as non-binary [20].

Many feedback comments expressed a strong desire for LGBTQ + health teaching, with some calling for it to be mandatory during the IMT programme, and others calling for it to be integrated into the IMT curriculum. Some participants were enthusiastic for teaching to be partly delivered by members of the LGBTQ + community as they felt it was important to hear “first hand patient experiences”.

While most of the feedback was positive, it is important to acknowledge the criticisms. One participant felt that LGBTQ + training is important during IMT, but "should not be priority". Another participant called for LGBTQ + training to be "carefully balanced against other learning needs" and that it should be implemented and "governed according to clinical need only".

## Strengths and limitations

Strengths of our study include the large sample size, and the fact that participants came from a diverse range of areas, both north and south London. Our research separately evaluated lesbian, gay, bisexual, and transgender health in certain questions, giving us a deeper insight into participants' understanding of these specific areas, something which is often omitted from studies in LGBTQ + health. The knowledge section presented three separate scenarios (lesbian woman, gay man, transgender man) while the confidence section examined sexual orientation and gender identity independently. Lastly, the knowledge section focused on areas of general medicine other than sexual health or mental health, which are often neglected in LGBTQ + medical education.

In terms of limitations, the generalisability of these results is restricted given the 40% response rate and the specific geographic location of this study. Participants were IMTs based in London, and consequently, one cannot draw accurate conclusions about levels of knowledge, confidence, and awareness among other groups of doctors, or doctors in other locations around the UK. Two potential explanations for the low response rate include the voluntary participation of the survey, and the fact that people may be reluctant to take surveys on “sensitive” topics (such as sexual orientation and gender identity). 23% of doctors in this survey identified as LGBTQ + , a higher proportion than expected in the general population, which could skew results. In the interests of time, and to avoid a lengthy survey, certain parameters were omitted, such as ethnicity (black, hispanic etc.), political affiliations (liberal, conservative, etc.), stage of Internal Medicine Training (IMT1, IMT2, IMT3), and attitudes towards LGBTQ + individuals.

### Implications for practice

#### Educational programs

Dedicated LGBTQ + educational programs are central in raising awareness among medical students and doctors about the healthcare disparities faced by LGBTQ + individuals and equipping them with the skills and knowledge to provide quality care. These programs should be designed by clinicians in conjunction with members of the LGTBQ + community. Constructivist educational activities should be prioritised, such as case-based discussions, patient interactions and role-play scenarios, as these promote active participation of learners which is key for cultural change [25]. Teaching should take place within a comfortable learning environment so that students feel safe to express opinions and critically examine various approaches to LGBTQ + healthcare, without feeling their views may be perceived as wrong or inappropriate. Educational programmes may be further enriched by embracing validated clinician self assessment tools; such as the Lesbian, Gay, Bisexual and Transgender Development of Clinical Skills assessment (LGBT-DOCSS); which allow trainees and clinicians to reflect upon their own knowledge and self-efficacy [26].

In designing education, we should avoid focusing solely on topics that are traditionally associated with LGBTQ + patients, such as sexual health. Links between the LGBTQ + community and general medical conditions such as cancer, cardiovascular disease, asthma and cognitive problems are less recognised, as evidenced by the results and feedback comments in our study. For example, the classic exam question of a gay male presenting with a new diagnosis of HIV or a sexually transmitted infection is useful to some degree, but it can lead to healthcare stereotyping [27] and fails to consider other associated medical conditions to which he is at risk. Our results show that doctors were particularly interested in teaching on transgender healthcare, especially terminology and relevant hormones. This is important to acknowledge, as we know that some clinicians feel under confident treating this group, and are not comfortable prescribing hormonal treatments [28].

Educators can be assisted in developing teaching materials by accessing support from partner organisations such as GLADD (The Association of LGBTQ + Doctors and Dentists) and the Fenway Health National LGBTQIA + Health Education Centre who can sign post to resources that providers might use, and support the building of networks that can share best practice in education [29, 30].

#### Integration into examinations

Integration of LGBTQ + health topics into formal assessments, both at undergraduate and postgraduate level, is important to promote an inclusive healthcare environment. Integration can be achieved by weaving LGBTQ + health topics into examinations, for example multiple choice questions and essay questions. Integration can also be achieved by swapping heterosexual or cisgender patients for LGBTQ + patients in clinical scenarios. For example, a traditional examination of an elderly patient with Parkinson’s disease can be swapped for an elderly transgender man with Parkinson’s disease. Most of the marks are still awarded for taking an appropriate neurological history and eliciting the correct signs on physical examination, but a small number of marks go towards appropriate communication, using correct pronouns and inclusive language. This encourages normalisation of these encounters and helps build confidence for doctors caring for these communities. For IMTs, LGBTQ + health topics could be integrated into the MRCPUK (Membership of Royal College of Physicians of the United Kingdom) examinations, required for successful progression to higher medical training. These topics should feature in the written sections, as well as the clinical sections (PACES) as suggested by participants in the feedback.

#### Curricular change

One of the most practical ways to ensure a topic is covered effectively during training is through integration into a curriculum. Currently, LGBTQ + health is not mandatory in British medical undergraduate curricula and studies demonstrate that coverage of LGBTQ + health topics at university level is very limited and extremely dependent on the staff in each university [16]. Growing voices are calling for this to be mandated with regulation from the General Medical Council [31]. Looking to the postgraduate setting, the situation is relatively similar with no mandatory coverage of LGBTQ + health topics for Foundation level or IMT doctors. The curriculum of the UK Foundation Programme asks for doctors to develop an understanding of "equality and diversity in health" but it fails to elaborate and does not specifically mention the LGBTQ + community, or other marginalised groups [32]. Likewise, the curriculum of Internal Medicine Training in the UK vaguely asks that "training bodies comply with equality and diversity standards", but again, fails to mention anything specific to the LGBTQ + communities [33]. LGBTQ + health training needs to be integrated into curricula, both undergraduate and postgraduate, with direct reference to sexuality and gender identity minorities, and their health inequalities. Furthermore, framework resources for reforming undergraduate curricula have already been published [34, 35], and these could be adapted for postgraduate curricula with relative ease.

#### Implications for research

Further studies are needed to evaluate levels of confidence and knowledge among other groups of clinicians. A comparative analysis could be done according to speciality (Psychiatrist, GP etc.), grade (registrar, consultant etc.) demographics, or geographic location, in an effort to identify factors associated with greater LGBTQ + health competency and disparities across various groups. Ideally, this would be carried out at a national level given that communities of LGBTQ + individuals are found throughout the country. Research should examine effective teaching methodologies to determine how best to integrate LGBTQ + topics into education and examinations. Longitudinal studies would help track changes in doctors' attitudes and behaviour over time, and examine competency before and after teaching interventions. In addition to targeting clinicians, future projects should explore the perspectives of LGBTQ + patients and their experiences in hospitals and clinics to determine the best ways of delivering high quality and healthcare.

## Conclusion

The results show there is a clear need for education on LGBTQ + health, given the variable levels of knowledge and confidence identified among Internal Medicine Trainees in London. A significant majority of participants have never received teaching on LGBTQ + health, although there exists a strong desire for this, particularly teaching on general medical issues facing LGBTQ + patients and transgender healthcare. Recommendations from our research include the creation of LGBTQ + educational programs, curricular change to include LGBTQ + topics, and the integration of LGBTQ + cases in postgraduate training and examinations for IMTs. There are very few published studies exploring competency in LGBTQ + health among doctors, with only one other in the United Kingdom, but none among British Internal Medicine doctors, making this study the first of its kind.

Our research highlights the necessity to address the educational needs of Internal Medicine Trainees in London in relation to LGBTQ + health, to improve patient experiences and outcomes, and to promote an inclusive healthcare environment for all.

## Data Availability

The authors confirm that the data supporting the findings of this study are available within the article and its supplementary materials.
